# NKG2D and DNAM-1 Ligands: Molecular Targets for NK Cell-Mediated Immunotherapeutic Intervention in Multiple Myeloma

**DOI:** 10.1155/2015/178698

**Published:** 2015-06-16

**Authors:** Cinzia Fionda, Alessandra Soriani, Alessandra Zingoni, Angela Santoni, Marco Cippitelli

**Affiliations:** Department of Molecular Medicine, Pasteur Cenci-Bolognetti Foundation Institute, Sapienza University of Rome, Viale Regina Elena 291, 00161 Rome, Italy

## Abstract

A pivotal strategy to improve NK cell-mediated antitumor activity involves the upregulation of activating ligands on tumor cells. Enhancement of NK cell-mediated recognition of multiple myeloma cells was reported by us and others showing increased surface expression of NKG2D and DNAM-1 ligands on tumor cells following treatment with a number of chemotherapeutic agents, such as genotoxic drugs or inhibitors of proteasome, histone deacetylases, GSK3, and HSP-90. These compounds have the capability to affect tumor survival but also to activate specific transduction pathways associated with the upregulation of different NK cell activating ligands on the tumor cells. Here, we will summarize and discuss the molecular pathways whereby these drugs can regulate the expression of NK cell activating ligands in multiple myeloma cells.

## 1. Introduction

Natural killer (NK) cells are important effectors in immune responses to tumors and viral infections whose effector function against target cells is generally related to their cytolytic activity. Moreover, by the secretion of different cytokines and chemokines, NK cells can also stimulate inflammatory responses and exert a control on adaptive immune responses [1, 2]. In this context, in the recent years, increased understanding of the mechanisms controlling NK cell activation has led to the development of therapeutic agents that can improve their responsiveness.

Multiple myeloma (MM) is a hematologic cancer characterized by clonal expansion of malignant plasma cells (PCs) that mainly reside in the bone marrow, able to interact with local microenvironment and bone marrow stromal cells (BMSCs) and these interactions are critical for survival and resistance to therapy [3]. Treatment strategies for MM have changed substantially in the past decade, and the use of autologous hematopoietic stem cell transplantation (HSCT) and the introduction of new drugs, such as bortezomib and immunomodulatory drugs (IMiDs), have significantly improved patients' survival [4–7]. Moreover, as an additional therapeutic strategy in young patients who experience early relapse or with very high risk features at diagnosis, allogeneic stem cell transplantation has been also considered, although often associated with significant transplantation-related morbidity or mortality [8]. However, despite advances in therapeutic strategies, MM remains an incurable disease (median survival around 4-5 years in adults) [9] and novel targeted therapies and synergistic combinations with appropriate antimyeloma agents are required.

Increasing evidences have shown that NK cells can elicit potent autologous and allogeneic responses to myeloma cells, strongly supporting their antitumor potential in response to immunomodulatory drugs or following stem cell transplantation [10–12]. Thus, an interesting strategy to treat this hematologic cancer could be to harness and boost NK cell antitumor activity; in particular, since impaired recognition of tumor cells represents a critical mechanism of immune evasion, an intriguing approach could be to make myeloma cells more susceptible to receptor-mediated recognition and killing by NK cells. Indeed, anticancer immune responses may contribute to the control of tumor progression after conventional chemotherapy, and different observations have indicated that a number of chemotherapeutic agents, or radiotherapy, can induce immune responses that result in immunogenic cancer cell death and/or immunostimulatory effects [13, 14].

Several studies have shown that the engagement of different activating receptors, such as the NKG2D (natural killer group 2, member D) and DNAX accessory molecule-1 (DNAM-1), plays an important role in the NK cell-mediated recognition and killing of MM cells [15–17]. Indeed, MM cells can express the NKG2D ligands MICA/B [15, 18], different UL16-binding proteins, the DNAM-1 ligands poliovirus receptor (PVR/CD155), and Nectin-2 [19].

A cogent example of the functional connection between chemotherapy and therapeutical immunomodulation is the finding that several genotoxic agents or drugs, such as inhibitors of proteasome, histone deacetylases, or the HSP-90 molecular chaperone, can increase the expression of NKG2D or DNAM-1 ligands, thus facilitating the activation of NKG2D/DNAM-1-expressing lymphocytes (e.g., NK cells, NKT cells, and CTLs) against tumor cells, including MM [17, 19–23].

Combinatorial therapies, in which NK cells represent one important mediator, may become a pivotal instrument for the development of future immunochemotherapeutical strategies.

Here, we provide a description of the molecular pathways activated by different pharmacological treatments used in the therapy of MM, aimed at enhancing NK cell-mediated tumor killing (Table 1).

## 2. DNA Damage Response Pathways

The DDR is a complex network of signal transduction pathways that has the ability to sense DNA damage leading to the arrest of the cell cycle, either transiently or permanently, through the activation of cell cycle checkpoints, and of specific DNA repair pathways. However, if the DNA damage is irreparable, cells can undergo apoptosis in order to prevent any damaged DNA progressing to deleterious mutations that would be passed down to its progeny [24, 25].

Tumor cells often display a defect in the DDR, associated with mutated or nonfunctional proteins involved in these pathways. In particular, MM malignant plasma cells are characterized by marked genomic abnormalities during tumor progression and have aberrant DNA repair pathways [26]. In this regard, monoclonal gammopathy of undetermined significance (MGUS) or MM patients exhibit a deregulated expression of cyclin D genes leading to a defect in the cell cycle check points [27]. Other genetic alterations can involve p53, ARF, NF-*κ*B, MYC, and KRAS genes, the gene products of which are critical in DNA repair pathways [27]. Several drugs used in MM therapy, such as melphalan, even at low doses, can induce DDR activation [28, 29]; in this context, we have recently contributed to delineating a link between the activation of DDR induced by chemotherapeutics and the transcriptional regulation of NKG2D and DNAM-1 ligands in MM. In particular, we observed the upregulation, at both protein and mRNA levels, of NKG2D and DNAM-1 ligand expression on MM cells (cell lines and primary malignant PCs) upon treatment with sublethal doses of commonly used genotoxic drugs such as melphalan and doxorubicin. In this context, the sublethal doses we used to treat the different MM cell lines corresponded to IC50 values 10 times lower, as previously described [19].

This effect was associated with the establishment of a chemotherapy-induced senescent phenotype characterized by permanent cell cycle arrest at the G_2_/M phase, flattened cell morphology, and positive senescence-associated *β*-galactosidase staining. Moreover, drug-induced ligand upregulation was dependent on the activity of the DDR protein kinases ATM/ATR and Chk-1/2 and on the E2F-1 transcription factor [19, 29]. Indeed, DDR activation leads to an ATM-dependent E2F1 accumulation, and a site for ATM/ATR phosphorylation in the amino terminus of E2F1 important for its stabilization has been identified [30]. Notably, at variance from our results showing that p53 is not involved in drug-induced ligand upregulation on malignant PCs, p53 involvement in ULBP1/2 upregulation on different human cancer cell lines was observed [31, 32], suggesting that p53 activity can exert opposite effects depending on the overall context of its activation.

We have also defined an important role for changes in the cellular redox state induced by sublethal doses of chemotherapy (melphalan, doxorubicin), in the control of DDR-dependent upregulation of ligand surface expression and gene transcriptional activity. Our observations, in accordance with much evidence indicating that DDR and oxidative stress are major determinants of cellular senescence, demonstrate that redox-dependent DDR activation plays a critical role for MM cell entry in premature senescence and is required for the preferential ligand upregulation on senescent cells [19, 29].

DDR is a tightly organized mechanism, governed by regulated protein-protein interactions and controlled also by a number of posttranslational modifications, including ubiquitination and sumoylation [33–36]. In this context, the disassembly, removal, and/or degradation of chromatin-associated DDR proteins represent an essential step in the double strand brakes (DSB) repair and postrepair processes and it is mostly coordinated by the ubiquitin-proteasome system (UPS) [37].

Bortezomib is a boronic acid 26S proteasome inhibitor which was approved by the Food and Drug Administration for the treatment of relapsed/refractory, relapsed, and newly diagnosed MM [38–40]. Interestingly, Jinushi and coworkers have demonstrated that bortezomib-mediated upregulation of MICA in myeloma cells required the activation of DDR, since shRNA silencing of ATM or Chk-2 blocked ligand induction [17]. Moreover, also DNAM-1 ligands expression is increased in response to bortezomib treatment both in primary malignant PCs and in MM cell lines [19].

Altogether, these data demonstrate a major role for the DDR pathways induced by genotoxic drugs or bortezomib, in the upregulation of NKG2D and DNAM-1 ligand on MM cells.

## 3. Hsp90 Inhibitors and Activation of HSF1

Hsp90 is a molecular chaperone able to directly bind, stabilize, and regulate the function of numerous client proteins, including many mediators of signal transduction and cell cycle progression [41]. Increased synthesis of Hsps is generally associated with stressful conditions which can cause protein denaturation/misfolding, but it is also a peculiarity of cancer cells whose proliferation depends on their capability to react to endogenous and exogenous stresses. In particular, Hsp90 is often overexpressed in different solid tumors and haematologic malignancies, such as MM, and can contribute to tumor cell survival by stabilizing many oncogenes and by interfering with apoptosis [42–44]. In MM, Hsp90 inhibition has been shown to affect multiple client proteins involved in pathways critical to tumor development and progression, angiogenesis, and osteoclastogenesis, such as IGF1 and IL-6 receptors, and PI3K/Akt, STAT3, and MAPK signaling pathways; moreover, upregulation of Hsp90 has been observed in MM cells interacting with BMSCs [45–47]. Accordingly, Hsp90 inhibitors have demonstrated potent antitumor activity in preclinical studies and several clinical trials of MM [46, 48, 49].

We found that treatment of MM cell lines with Hsp90 inhibitors [radicicol or 17-allylaminogeldanamycin (17AAG)] results in a significant upregulation of MICA and MICB expression, rendering these cells more efficient to activate NK cell degranulation [22]. To identify possible mechanisms underlying NKG2DL upregulation, we focused our attention on two different cellular responses induced by Hsp90 inhibitors: the “heat shock response” (HSR) and the “unfolded protein response” (UPR). In this regard, Hsp90 is considered a key factor in the regulation of HSF1, a transcription factor involved in the induction of the HSR. Under nonstress conditions, Hsp90 together with other components of the Hsp90 chaperone machinery interacts with HSF1 and represses its transcriptional activity [50]. Moreover, HSF1 is a known regulator of chaperone genes and its activation induces increased expression of Hsp90, thus providing an autoregulatory mechanism for its own inhibition. However, acute stress-induced HSF1 controls the expression of different target genes. In this regard, this transcription factor has been shown to mediate MICA and MICB promoter activation by heat shock [51]. Exposure of MM cells to Hsp90 inhibitors, able to block the HSF1/Hsp90 autoregulatory loop, induces the release, nuclear translocation, and binding of HSF1 to a heat shock response element (HSRE) on MICA/MICB promoters; moreover, knockdown of HSF1 using small hairpin RNA interference blocks these effects, indicating that HSF1 activation is essential for MICA and MICB upregulation by Radicicol and 17AAG [22]. The UPR consists in the accumulation of misfolded proteins and the induction of the ER stress, leading to the activation of complex signaling and transcriptional pathways [52, 53]. However, UPR activation, as revealed by XBP1 and CHOP presence, is weakly induced or inhibited by Hsp90 inhibitors in a time- and dose-dependent manner in MM cells [48]; moreover, treatment of MM cells with two classical ER stress inducers, such as tunicamycin or thapsigargin, failed to modulate MICA or MICB expression, suggesting that UPR activation, per se, is not sufficient to enhance levels of these ligands and that it is not involved in their regulation by drugs targeting Hsp90 [22].

## 4. GSK3 Inhibitors and STAT3

The serine/threonine kinase GSK3, for years considered only for its role in glycogen metabolism, now is a known component of diverse cellular signaling pathways involved in the regulation of protein synthesis, cell motility, proliferation, and survival [54–57]. Moreover, GSK3 has been shown to have a positive role in cancer and its pharmacological inhibition holds promise for therapeutic intervention in several solid and hematologic tumors [58]. Interestingly, this protein kinase has emerged as a critical molecule in the pathogenesis of MM [59–61]. Studies on the expression and function of GSK3 in MM cells have reported an abundant expression of the two GSK3 subunits, *α* and *β*, and identified GSK3*α* as the prevailing active isoform. Indeed, GSK3 inhibitors can induce growth arrest or apoptosis in MM cell lines and can enhance the anti-MM cytotoxic effect of bortezomib, by modulating critical signaling pathways in these cells such as the forkhead transcription factors FHRL1 and FKHR, *β*-catenin, and extracellular signal-regulated kinase- (ERK-) 1/2 kinases. Moreover, GSK3-mediated phosphorylation can stimulate the activity of different transcription factors sustaining MM cell growth, such as NF-*κ*B and Maf [62]. In this regard, administration of the GSK3 inhibitor [(2′Z,3′E)-6-bromoindirubin-3′-oxime] (BIO) in models of myeloma bone disease has been shown to ameliorate bone destruction associated with MM progression, enhancing the osteogenesis in mesenchymal stem cells and, in parallel, inducing regression of the tumor [61].

We found that different drugs targeting the GSK3 kinase [e.g., lithium chloride (LiCl), SB216763 (SB21), or BIO] can upregulate both MICA protein surface and mRNA expression in MM cells, with little or no effects on MICB and PVR expression [63]; moreover, exposure to GSK3 inhibitors renders myeloma cells more susceptible to NK cell-mediated cytotoxicity. Intriguingly, we also showed that STAT3 repression plays a critical role in the upregulation of MICA expression induced by GSK3 inhibitors [63]. Similarly, a previous study had demonstrated that STAT3 is a negative regulator of MICA transcription in different cancer cell lines [64]; moreover, GSK3 activity has been shown to positively influence the tyrosine^705^ (Tyr^705^) phosphorylation and DNA-binding activity of STAT3 in response to different cytokines, and inhibition of this kinase could significantly modulate the expression of STAT3-regulated genes [65]. In this context, we showed that treatment of MM cells with the STAT3 inhibitor STA-21 or with the JAK2-specific inhibitor AG490 can increase MICA expression, thus confirming the repressive action of STAT3 on this gene also in this type of cancer cells. Indeed, our data showed that treatment of MM cells with drugs targeting GSK3 led to a marked reduction of the constitutive STAT3 phosphorylation in Tyr^705^ and its binding to the promoter fragment encompassing a repressive MICA/STAT3 response element. Moreover, overexpression of a constitutively active mutant form of STAT3 significantly inhibited MICA upregulation by GSK3 inhibitors, indicating that one of the mechanisms involved in GSK3-mediated regulation of* mica* gene expression could be related to the transcriptional activity of its promoter, where basal repression mediated by active STAT3 can be released by GSK3 inhibition. The mechanisms underlying MICA repression by STAT3 remain to be explored. STAT3 has been shown to inhibit certain tumor suppressor genes via epigenetic modifications, such as CpG island methylation [66, 67]. In this regard, NKG2D ligand expression by histone deacetylase (HDAC) or DNA methylation inhibitors was described in different cancer cells, suggesting that chromatin modifications can control the basal expression of these ligands on tumor cells [21, 68–71]. These findings suggest that epigenetic modifications likely can contribute to STAT3-dependent repression of* mica* promoter activity; however, additional experiments are needed to better analyze this hypothesis.

## 5. Histone Deacetylase Inhibitors (HDACi)

Histone deacetylase inhibitors (HDACi) are a novel class of anticancer agents undergoing evaluation in clinical trials for the potential treatment of patients with different cancers, including hematopoietic malignancies and MM. Indeed, HDACi are able to induce increased acetylation of DNA-associated histone proteins, leading to cell cycle arrest, differentiation, and/or apoptosis in a wide range of malignant cells [21, 72, 73]. A large body of evidence shows that treatment of different type of tumor cells with HDACi leads to the upregulation of NKG2D ligand surface expression resulting in a significant increase of NK cell-mediated lysis of tumor cells [21, 68–70]. In line with these results, a recent study performed on human MM cells demonstrates that valproic acid (VPA), a molecule originally described as an antiepileptic and then demonstrated to inhibit HDACs inducing antineoplastic activity), is able to enhance the expression of the NKG2D ligands MICA/B and ULBP-2 with a mechanism dependent on the activation of constitutively phosphorylated ERK [23]. Interestingly, treatment of MM cells with VPA increased the expression of pERK-1 and reduced pERK-2 levels; in this regard, although the reason for the preferential phosphorylation of ERK-1 in VPA-treated myeloma cells was not investigated, the underlying mechanism might be explained by loss of competition between ERKs for their binding/activation by mitogen-activated protein kinases [23].

## 6. Possible Cross Talk between Drug-Activated Pathways Inducing NKG2D and DNAM-1 Ligand Expression in MM Cells

Integration of different pathways regulating the expression of NKG2D and DNAM-1 ligand in MM cells could be beneficial to enhance NK cell recognition of tumor target cells. Moreover, treatment of MM implies the simultaneous administration of different pharmacologic agents, so it would be helpful to understand how and if multiple signaling events triggered by different drugs could affect surface levels of NK cell activating ligands; however, very few data are available about the potential effects of combined therapies on the expression of these molecules on MM cells.

Several studies have described synergistic antimyeloma effect of different pharmacologic agents including GSK3 or Hsp90 inhibitors with bortezomib [60, 74, 75] or with melphalan and doxorubicin [76, 77].

In this regard, we have recently investigated the possibility of cross talk between pathways induced by chemotherapeutic agents. The cooperation between GSK3 inhibition and genotoxic agents in the induction of MICA expression has been investigated in MM cells. We observed that GSK3 inhibition can cooperate with drug-activated DDR to increase MICA expression, since treatment with melphalan increased the expression of MICA and this upregulation was further enhanced in the presence of LiCl. This cooperation may be due to different and independent cellular events triggered by two drugs; alternatively, treatment with GSK3 inhibitors may facilitate the action of melphalan and/or vice versa. Interestingly, STAT3 constitutive activation was shown to prevent the induction of MICA following genotoxic stress [64]. These observations suggest that constitutive activation of this transcription factor in MM cells may interfere with pathways triggered by DDR, increasing the threshold for optimal activation; it could be speculated that GSK3 inhibition may favor MICA upregulation after melphalan treatment by reducing the repressive activity of STAT3. In addition, GSK3 has been shown to regulate E2F1 activity by means of direct and indirect mechanisms. In particular, independently by its kinase activity, GSK3 has been found to physically interact with the transactivation domain of E2F1 and to inhibit its transcriptional activity [78, 79]. These observations can suggest E2F1 activation as a possible point of convergence between DDR and GSK/STAT3, resulting in further increase of activating ligand expression.

**Figure 1:**
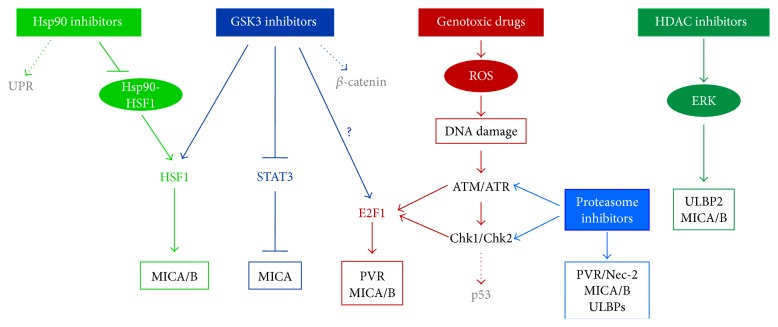
Drug-activated pathways regulating NK cell activating ligand expression. Hsp90 inhibitors regulate MICA/B expression via HSF1 activation. Drugs targeting GSK-3 repress STAT3 leading to MICA upregulation. Genotoxic drugs induce the expression of NKG2D or DNAM-1 ligands following the activation of DDR-dependent E2F1 transcription factor. Proteasome inhibitors induce MICA expression via ATM and Chk-2 activation. HDAC inhibitors increase MICA/B and ULBP2 levels with a mechanism dependent on ERK activation. Molecules or pathways not involved in the regulation of these ligands are indicated in grey color and with dotted raw.

Despite the lack of data about the combined use of other drugs and NK cell ligand expression in MM cells, the fact that a growing number of studies described synergic antimyeloma effects of these pharmacologic agents strongly suggests that this aspect should be better investigated (Figure 1).

## 7. Chemotherapy and NKG2D Ligand Shedding: A Double Edge Sword?

The release of soluble NKG2D ligands has been suggested to be a major mechanism of tumor cell evasion from NKG2D-mediated immunosurveillance. As a matter of fact, soluble forms of NKG2D ligands are present in the serum of MM patients [17, 80] and other types of malignancies; in this regard, their levels correlate with tumor stage and metastasis and with reduced expression of NKG2D on NK cells and other cytotoxic lymphocytes [81, 82]. Soluble NKG2D ligands can be released through metalloproteinase-mediated cleavage, exosome secretion, or alternative splicing; however, little is known about the effect of different chemotherapeutic drugs on NKG2D ligands shedding. Although Kohga and coworkers have shown that epirubicin can reduce the shedding of MICA in epatocarcinoma cell lines [83], a large body of evidence denotes that conditions causing cellular stress, including chemotherapeutic agents and ROS, can lead to increased metalloproteinase-mediated release of cell surface molecules, including NKG2D ligands [84–87]. Interestingly, Huang and coworkers have shown that combination of valproate, known to upregulate cell surface MICA/B, and metalloproteinase inhibitors was found to significantly stabilize cell surface MICA/B on ovarian carcinoma cells and to enhance* in vivo* the efficacy of immune cell therapy [88]. Moreover, in osteosarcoma cells, valproate treatment can downregulate MMP9 expression and thereby upregulate cell surface MICA/B expression, inhibiting the release of soluble forms of these ligands [89].

Thus, metalloproteinases implicated in NKG2D ligands shedding could be targeted in novel therapeutic schemes to regulate the escape of malignant cells from stress-elicited immune responses. In this regard, additional studies will be needed to better analyze NKG2D ligands shedding and the pathways involved in its regulation by chemotherapeutic agents in MM.

## 8. The Other Side of the Coin: How Pharmacologic Treatments Can Impact NK Cells

The setting of therapeutic approaches, based on chemotherapy-induced sensitization of tumor cells to NK cell-mediated cytotoxicity, should always consider the possible drug-induced effects when chemotherapy and the activity of NK cell-mediated actions are needed together. In this regard, standard and high dose chemotherapeutic regimens for malignancies can inhibit the activity of the immune system and also significantly decrease NK cell-mediated killing [90]. However, the effects of their immunomodulatory potential could be changed and improved by using different doses and schedules. In this context, we observed that treatment of NK cells, with sublethal concentrations of doxorubicin, does not affect the ability of NK cells to degranulate in response to MM cells, as well as the expression of NKG2D and DNAM-1 and ability to produce IFN-*γ* [29]. On the contrary, the activity of GSK3 kinase has been shown to modulate specific functions of NK cells; inhibition of its activity can increase cytokine secretion and cytotoxicity, possibly due to nuclear translocation of functional *β*-catenin [91]. Similarly, in different* in vivo* models of hematologic cancer, loss of STAT3 in NK cells enhances tumor surveillance by increasing their cytolytic activity [92]. Thus, the observations summarized above about the interplay GSK3/STAT3 and MICA expression in MM cells would be supported also by the additional information that inhibition of this kinase could directly enhance the activity of NK cells against the tumor.

Hsp90 is critical for regulation of phenotype and functional activity of NK cells. How, after Hsp90 inhibition, NK cells display decreased activating receptor expression which correlate with a downregulation of their cytolytic activity against tumor cells has been described [93]. Likewise, NK cell effector functions can be compromised following treatment with HDACi or bortezomib. Indeed, HDACi (e.g., vorinostat, trichostatin A, valproic acid, and apicidin) exert their suppressive effect on both resting and activated NK cells and at doses not affecting NK cell vitality, with reduced levels of the activating receptors NKG2D and NCRs [94, 95]. Moreover, these drugs can also downregulate ligands for NK cells-activating receptors, such as B7-H6 (a ligand for NKp30), and impair tumor cell recognition by NK cells [96].

Cytotoxic effects of bortezomib on immune-competent cells have also been observed. In this regard, bortezomib can trigger apoptosis and disrupt NKp46-dependent cytotoxicity in primary human NK cells [97]. Moreover, bortezomib can inhibit surface expression of TRAIL in activated human NK cells [98].

The development of combined cytoprotective strategies to prevent the adverse effects of bortezomib on NK cells, together with the use of adoptively transferred NK cells, will be needed to enable a more efficient use of this important class of drugs in MM patients.

## 9. Concluding Remarks

A number of experimental studies have shown that NK cells have the ability to eliminate cancer cells; in this context, the activity of NK cells can be exploited in therapeutic strategies against different cancers. As discussed above, a number of chemotherapy-induced molecular pathways can upregulate NKG2D and DNAM-1 activating ligands, able to increase activation and cytotoxic responses of NK cells toward MM. Future preclinical research and the standardization of combined therapeutic protocols using anticancer agents and NK cells should be encouraged to promote effective therapeutic immune responses to MM.

## Figures and Tables

**Table 1:** Drug-induced pathways and molecular targets associated with the upregulation of NKG2D and DNAM-1L expression on MM cells.

Drug	Pathway/molecular target	Ligands	References
*Genotoxic agents* Doxorubicin Melphalan	ROS-dependent DDR	MICA/B, ULPB1-3 PVR, Nectin-2	Soriani et al., 2009 [19]; Soriani et al., 2014 [29]

*GSK3 inhibitors* LiCl BIO SB21	STAT3 inhibition	MICA	Fionda et al., 2013 [63]

*Proteasome inhibitor* Bortezomib	DDR	MICA, ULBP1-3, PVR, Nectin-2	Jinushi et al., 2008 [17] Soriani et al., 2009 [19]

*Histone deacetylase inhibitor* Valproic acid	ERK	MICA/B ULBP-2	Wu et al., 2012 [23]

*Hsp90 inhibitors* Radicicol, 17-AAG	HSR	MICA/B	Fionda et al., 2009 [22]

DDR: DNA damage response; ROS: reactive oxygen species.

HSR: heat shock response; ERK: extracellular signal-regulated kinase.

## Abbreviations

DDR: DNA damage response
DNAM-1: DNAX accessory molecule-1
MM: Multiple myeloma
MMP: Matrix metalloprotease
NCR: Natural cytotoxicity receptors
NKG2D: NK group 2D
PVR: Poliovirus receptor-CD155
ROS: Reactive oxygen species.
